# Region-Specific Microstructure in the Neonatal Ventricles of a Porcine Model

**DOI:** 10.1007/s10439-018-2089-4

**Published:** 2018-07-16

**Authors:** F. Ahmad, S. Soe, N. White, R. Johnston, I. Khan, J. Liao, M. Jones, R. Prabhu, I. Maconochie, Peter Theobald

**Affiliations:** 1 School of Engineering, Cardiff University, Cardiff, UK; 2 School of Optometry and Vision Sciences, Cardiff University, Cardiff, UK; 3 College of Engineering, Swansea University, Swansea, UK; 4 Swansea University Medical School, Swansea University, Swansea, UK; 5 Department of Bioengineering, The University of Texas at Arlington, Arlington, USA; 6 Department of Biological Engineering, Centre for Advanced Vehicular Systems, Mississippi State University, Mississippi, USA; 7 Imperial College NHS Healthcare Trust, London, UK

**Keywords:** Diffusion tensor magnetic resonance imaging, Two-photon excitation, Second-harmonic generation, Cardiomyocyte architecture, Fibre orientation, Fibre dispersion, Neonatal heart structure

## Abstract

The neonate transitions from placenta-derived oxygen, to supply from the pulmonary system, moments after birth. This requires a series of structural developments to divert more blood through the right heart and onto the lungs, with the tissue quickly remodelling to the changing ventricular workload. In some cases, however, the heart structure does not fully develop causing poor circulation and inefficient oxygenation, which is associated with an increase in mortality and morbidity. This study focuses on developing an enhanced knowledge of the 1-day old heart, quantifying the region-specific microstructural parameters of the tissue. This will enable more accurate mathematical and computational simulations of the young heart. Hearts were dissected from 12, 1-day-old deceased Yorkshire piglets (mass: 2.1–2.4 kg, length: 0.38–0.51 m), acquired from a breeding farm. Evans blue dye was used to label the heart equator and to demarcate the left and right ventricle free walls. Two hearts were used for three-dimensional diffusion-tensor magnetic resonance imaging, to quantify the fractional anisotropy (FA). The remaining hearts were used for two-photon excited fluorescence and second-harmonic generation microscopy, to quantify the cardiomyocyte and collagen fibril structures within the anterior and posterior aspects of the right and left ventricles. FA varied significantly across both ventricles, with the greatest in the equatorial region, followed by the base and apex. The FA in each right ventricular region was statistically greater than that in the left. Cardiomyocyte and collagen fibre rotation was greatest in the anterior wall of both ventricles, with less dispersion when compared to the posterior walls. In defining these key parameters, this study provides a valuable insight into the 1-day-old heart that will provide a valuable platform for further investigation the normal and abnormal heart using mathematical and computational models.

**Electronic supplementary material:**

The online version of this article (10.1007/s10439-018-2089-4) contains supplementary material, which is available to authorized users.

## Introduction

Within moments of birth the human body transitions from placenta-based oxygenation, to a lung-derived supply. This necessitates a complex series of self-regulated structural and functional changes, including diversion of blood through the right ventricle and to the lungs. In some cases, however, the heart structure does not fully develop causing poor circulation and inefficient oxygenation, which is associated with an increase in mortality and morbidity. Congenital heart disease (CHD) describes such abnormalities within the heart structure and is the most common birth defect, affecting 9 in 1000 births and causes 10% mortality before school-age.^12^,^27^ This study focuses on developing an enhanced understanding of the 1-day-old heart. This will be achieved by quantifying the region-specific microstructural parameters of the tissue and will ultimately enable improved accuracy when mathematically and computationally simulating conditions including CHD.

The heart undergoes rapid structural and functional change during the first few hours of life, meaning there are striking anatomical variations between even the full-term foetal and 1-day old heart. In the former, oxygenated blood returns to the heart from the umbilicus, entering the right atrium *via* the inferior vena cava, before the majority is shunted through the foramen ovale to the left heart. Any remaining input, combined with the superior vena cava return that also enters the right atrium, flows through the tricuspid valve. On ejection from the right ventricle, only 10–15% reaches the pulmonary circulatory system, with the majority diverted away from the lungs through the ductus arteriosus. This achieves equalised pressure in the right and left ventricles (60 mmHg) and in the aorta and pulmonary artery (60/40 mmHg),^26^ meaning the foetal right and left ventricular walls have similar thickness.^15^ Ventilation of the lungs at birth, soon followed by complete closure of the ductus arteriosus,^25^ creates a dramatic increase in flow through the pulmonary artery. A concurrent decrease in vascular resistance, however, causes an overall reduction in pulmonary arterial and right ventricle pressures (to 30/15 and 30 mmHg, respectively). The right atrial pressure reduces from 3 to 0 mm/Hg. The aortic and left ventricle pressures increase (to 75/50 and 75 mmHg respectively), establishing the pressure differential and initiating wall thickening that leads to the familiar variation between the right and left heart.

Incomplete heart development is a predominant cause of CHD, with surgical integration of an implant a common approach to septal defect repair.^22^,^32^,^35^ A poor understanding of the neonatal tissue behaviour has, however, been previously recognised as a contributing factor to the failure rates of such grafts, with enhanced data having, by implication, the potential to positively influence CHD mortality and morbidity.^14^ Novel tissue engineered solutions also offer potential for repair^34^; however, an ability to accurately simulate the likely outcome of these interventions would assist in identifying an appropriate scaffold material.^14^ This study focuses, therefore, on quantifying the microstructural parameters that underpins this behaviour.

Mature myocardium tissue is known to have a highly organised structure, which aids in achieving optimal functionality. The extracellular matrix (ECM) provides a three-dimensional structure and includes collagen fibres and cardiomyocytes, both of which significantly influence the gross biomechanical properties of cardiac tissue. Neither the cardiomyocytes nor collagen fibres are perfectly aligned in the mature myocardial tissue,^13^ with their ‘fibre direction’ (i.e. orientation) and ‘angular dispersion’ (i.e. disorganisation) essential to maintaining myocardial stiffness and anisotropy during the cardiac cycle. This direction and dispersion also influence the passive and active behaviour of adult myocardial tissue.^10^,^20^,^21^

Whilst the structure of the 1-day old heart is unknown, bio-mechanical tests have demonstrated it shares key traits with mature tissue: it is non-linear, anisotropic, viscoelastic and heterogeneous. The 1-day old porcine cardiac tissue exhibits one-half the stiffness of mature porcine tissue in uniaxial extension testing, one-third in biaxial extension testing, and one-fourth stiffness in simple shear testing vs. animal mature porcine tissue; hence, whilst the overall behaviour of 1-day old tissue is similar to mature, the neonatal tissue is likely to have a different microstructure.^11^

Current understanding of the young cardiac tissue microstructure is limited to qualitative data, derived from histological analysis of the left ventricle.^1^,^8^,^31^ Emerging three-dimensional techniques are now providing opportunity for quantitative assessment. Two-photon-excited fluorescence (TPEF) and second harmonic generation (SHG) enable optical sectioning of relatively thick tissue samples.^17^,^18^ The former can image elastin and cardiomyocytes by exciting endogenous fluorophores, whilst SHG provides a deeper insight into those molecules lacking a centre of symmetry (e.g., collagen, microtubules and myosin).^5^,^6^ Used in tandem, the two techniques provide a microscopic, 3D representation of the interplay between key proteins.^7^ Diffusion tensor magnetic resonance imaging (DT-MRI) provides a platform for assessing the fractional anisotropy (FA), quantifying the water diffusion anisotropy and thereby describing the directional coherence of cardiomyocyte orientation.^37^,^38^

This study will use these techniques to characterise the microstructure of 1-day old cardiac porcine tissue, focussing on the potentially different structures in the anterior and posterior aspects of the left and right ventricle ‘free-walls’. These data will be of value to scientists, bioengineers and mathematicians who are all investigating heart conditions in the young.

## Materials and Methods

### Materials

Twelve, 1-day-old deceased Yorkshire piglets (mass: 2.1–2.4 kg, length: 0.38–0.51 m) were acquired from a breeding farm. The piglets most likely died from hypoxia shortly after birth, with all piglets and heart development consistent with being born live. Piglets were collected within hours of their death and transported at 4 °C to a Cardiff University laboratory. Hearts were promptly harvested and carefully inspected for any macroscopic damage or disruption, before being stored in Ringer’s solution (Oxoid; Thermo Scientific, UK). Evans blue dye was used to label the heart equator and to demarcate the RVFW and LVFW.

**Figure 1:**
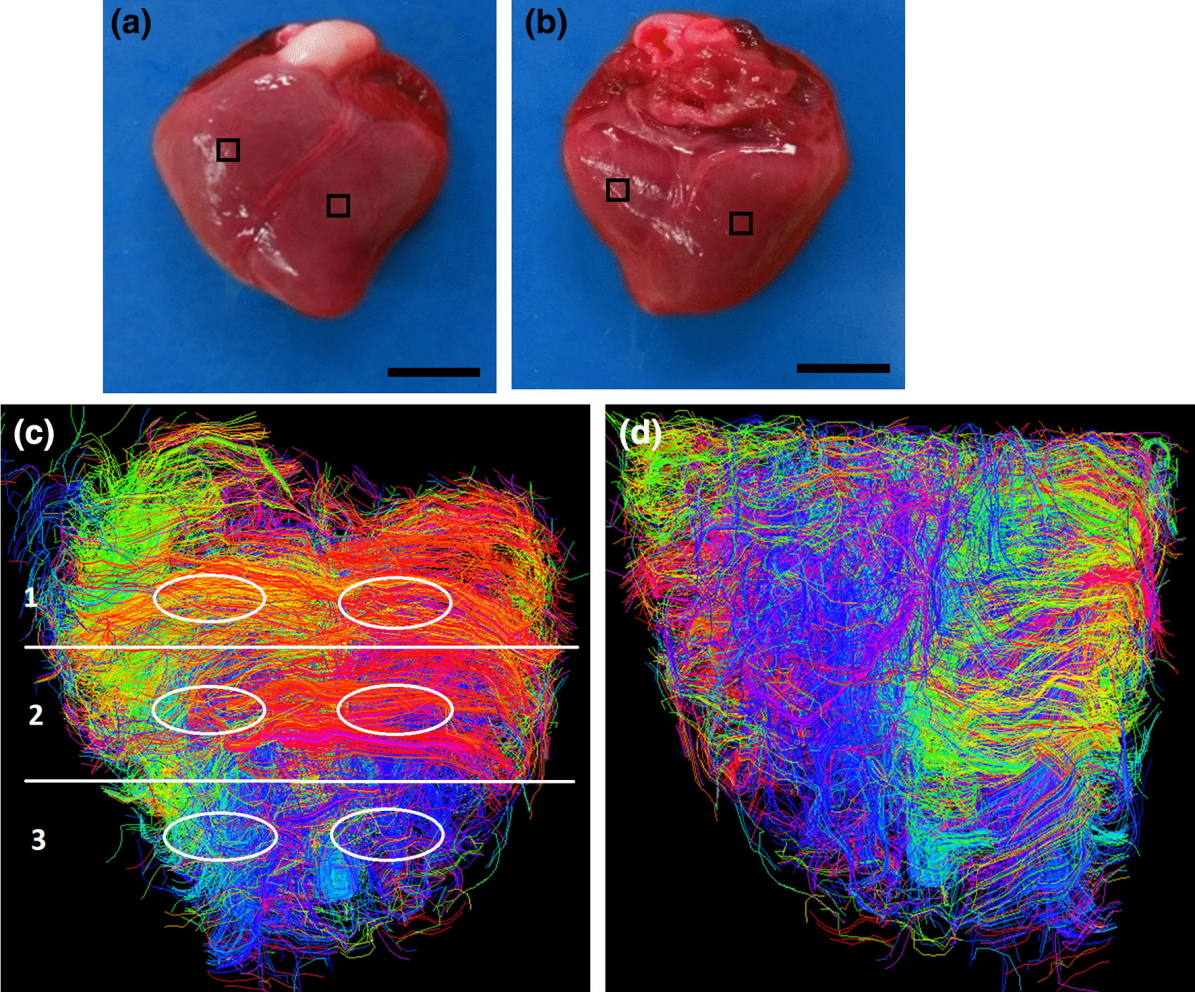
The anterior (a) and posterior (b) aspects of one-day-old neonatal porcine heart. Post-processed DT-MRIs at 25% fibre density, enabling visualisation of the fibre orientation on the anterior (c) and posterior (d) surface. The regions of interest used to calculate the fractional anisotropy (FA) for the base identified as: (1) base, (2) equator, and (3) apex. Scale bar = 8 mm.

Two hearts were used for DT-MRI scans. Five hearts were used for in-plane (*x*, *y*) TPEF/SHG analysis. A reference axis was defined passing through the apex and base, with the edge of a 2 × 2 mm square cutter kept parallel to this axis when dissecting tissue samples, taken through the ventricle walls. Samples (4 each from the anterior and posterior, LVFWs and RVFWs) were collected from around the equator of each heart (Figs. 1a and 1b), making a total of 20 samples for in-plane analysis. A further twenty samples were collected from an additional five hearts using an identical technique, for out-plane (*x*, *z*) analysis. All samples were immersed in Ringer’s solution throughout, to minimise tissue shrinkage.

### Methods

#### DT–MRI Image Acquisition and Analysis

Diffusion-weighted images were acquired with a Bruker MRI scanner, 9.4 Tesla small bore MRI and magnetic resonance spectroscopy (MRS) system. Hearts were placed in conical plastic centrifuge tubes filled with Ringer’s solution. A data volume of 96 × 96 × 36 mm was acquired with a voxel size of 1.17 × 1.17 × 2.6 mm. The GE diffusion tensor imaging protocol was used, with two b values (0, 1000) and 55 gradient directions.^38^

3D Slicer software plug-in ‘SlicerDMRI’ was used to perform unscented Kalman filter tractography and scalar measurements. Diffusion-weighted images were used to calculate the diffusion tensor. The diffusion tensor D in each voxel was visualised as a diffusion ellipsoid. The eigenvectors were used to define the directions of the principal axes, and the ellipsoidal proportional to the square root of the eigenvalues. The size and shape of the diffusion tensor were described by rotationally invariant eigenvalues *λ*_1_, *λ*_2_, *λ*_3_. Diffusion tensor imaging was used to evaluate the trace and fractional anisotropy. Trace (D) and fractional anisotropy (FA) are scaler measures, intrinsic to tissue and are independent of fibre orientation and diffusion sensitizing gradient directions. Trace was used to calculate the size of the tensor, whereas fractional anisotropy characterised the shape (degree of ‘out of roundness’) of the diffusion ellipsoid, ranging from 0 (low FA) to 1 (high FA). The Tractography Interactive seeding module was then used on the FA map to track the cardiomyocytes. Undesirable tracks were removed to obtain the required heart profile (Supplementary Figure A.1). Finally, the regions of interest (ROIs) were selected (i.e. base, equator and apex of RVFW and LVFW) to obtain the regional FA. This method is consistent with that described elsewhere.^19^

#### TPEF/SHG Image Acquisition and Analysis

TPEF/SHG images were acquired by non-linear microscopy (NLM), using a laser scanning microscope (LSM880 NLO, Carl Zeiss, Ltd. Cambridge, UK) equipped with an ultrafast-pulsed near-infrared (NIR) TiS laser illumination system (Chameleon Vision II, Coherent Lasers, Cambridge, UK). Laser excitation at 900 nm and an approximate 140 fs pulse width were used for all NLM imaging, which was passed to the specimen and separated from returning emissions by a 690 nm short-pass primary dichroic reflector. Backwards propagating TPEF and SHG light from the specimen was collected by the objective and detected in the reflected light (epi-) pathway of the microscope, using the internal spectrometer to select the desired wavelengths. SHG (at half the excitation wavelength) was detected at 450 ± 10 nm and TPEF at all wavelengths longer than 470 nm.

Two channel (TPEF and SHG), 8-bit images were acquired simultaneously at serial focal positions to build up a 3D stack of optical sections collected at 1.52 *µ*s. These comprised in-plane (*x*, *y*) and out-plane (*x*, *z*) stacks, with total volume 425 × 425 × 202 *µ*m, and 425 × 425 × 1022 *µ*m intervals, respectively. Each line of every 2D optical section was scanned 8 times and the average signal recorded. The laser power during the acquisition of deeper images was automatically increased following a pre-set pattern, to compensate for the light-scattering reducing the illumination.

Tissue samples were fixed into a plastic petri dish using medical glue and immersed in Ringers solution (Oxoid; Thermo Scientific, UK), into which was dipped the objective lens for NLM imaging. All NLM imaging was performed using this technique (W Plan-Apochromat 20x/1.0NA, Carl Zeiss). Fiji/Image J (NIH, USA) software was then used to perform quantitative analysis on TPEF/SHG image stacks. In-plane (*x*, *y*) and out-plane (*x*, *z*) images were pre-processed in three dimensions using selected computational filters (unsharp mask, Gaussian blur 3D and Kuwahara), prior to these ‘stacks’ being analysed using the Fourier components analysis method.^16^ (‘stacks’ are typically termed ‘*z*-stacks’; however, in considering out-plane images this term may become misleading, as it would instead be a ‘*y*-stack’, so the generic term ‘stack’ is adopted hereafter). This approach enabled quantification of the collagen and cardiomyocyte distributions, relative to the stack depth. Using the ImageJ plug-in ‘Directionality’ (https://imagej.net/Directionality), data from all images within the stack were used to generate a histogram. The peak was then fitted to a Gaussian function, enabling identification of the ‘preferred’ fibre orientation direction, a method consistent with other studies.^23^,^24^ The output comprised: (1) the preferred fibre orientation direction (°), defined by the centre of the Gaussian distribution; (2) the angular dispersion (°), defined as the standard deviation (std) of the Gaussian distribution; (3) the amount parameter, defined as the sum of the histogram from minus 1 std to plus 1 std, divided by the total sum of the histogram; (4) goodness of fit (*R*^2^). TPEF/SHG images with *R*^2^ > 0.8 were used for further analyses, an identical threshold to that adopted elsewhere.^28^

#### Statistical Analysis

All values were reported as mean ± SD, with statistical significance given to values less than 0.05. Variations in the mean in-plane and out-plane cardiomyocyte rotation and dispersion were compared between the anterior and posterior walls within, and between, the two ventricles, using a one-way ANOVA and Tukey HSD *post hoc* test. An identical approach was also used to assess the average collagen fibrils rotation and dispersion. All statistical analyses were performed in SPSS 20.0.

## Results

### DT–MRI Analysis

DT-MRI enabled observation of the gross anatomy of the neonatal heart, with the anterior exhibiting greater curvature than posterior surfaces.

#### LVFW

FA varied significantly (*p* < 0.05) across the LVFW, being greatest in the equatorial region (0.75), followed by the base (0.72) and apex (0.70) (Table 1, Figs. 1c and 1d). Cardiomyocyte density appeared greatest in the lower base and equatorial regions (Supplementary Figs. 2a–2f), being aligned near-horizontally in the anterior wall (relative to the defined vertical axis of the heart, passing through the apex and base). Posteriorly, fibres were near-diagonally aligned when. Cardiomyocytes were predominantly aligned in parallel towards the lower base and equatorial regions, though alignment appeared weaker in the apex.

**Table 1:** The total number of fibre tracks and regional fractional anisotropy (FA) in the anterior LVFW and RVFW.

ROIs	Fibre number	FA
LVFW		
Base	795	0.72 ± 0.06^a,b^
Equator	330	0.75 ± 0.04^a,b^
Apex	1284	0.70 ± 0.03^a,b^
RVFW		
Base	339	0.74 ± 0.05^a,b^
Equator	169	0.77 ± 0.03^a,b^
Apex	789	0.71 ± 0.06^a,b^

Results are expressed as mean ± SD

^a^One-way analysis of variance (ANOVA) revealed statistical significance between the base, equator and apex *within* the ventricle *p* < 0.05

^b^One-way analysis of variance (ANOVA) revealed statistical significance between equivalent regions *across* the ventricles *p* < 0.05

##### RVFW

The equatorial region was again the region of highest FA (0.77) within the RVFW. This was again significantly greater (*p* < 0.05) than the base (0.74), which too was greater than the apex (0.71) (Table 1). The FA in each region was also statistically greater than that in the LVFW (Table 1). Greater cardiomyocyte density was again observed in the lower base and equatorial regions, though in the posterior surface they were aligned horizontally—and so consistent with the anterior aspect, unlike the LVFW (Supplementary Figs. 2a–2f).

##### TPEF/SHG Analyses

**Figure 2:**
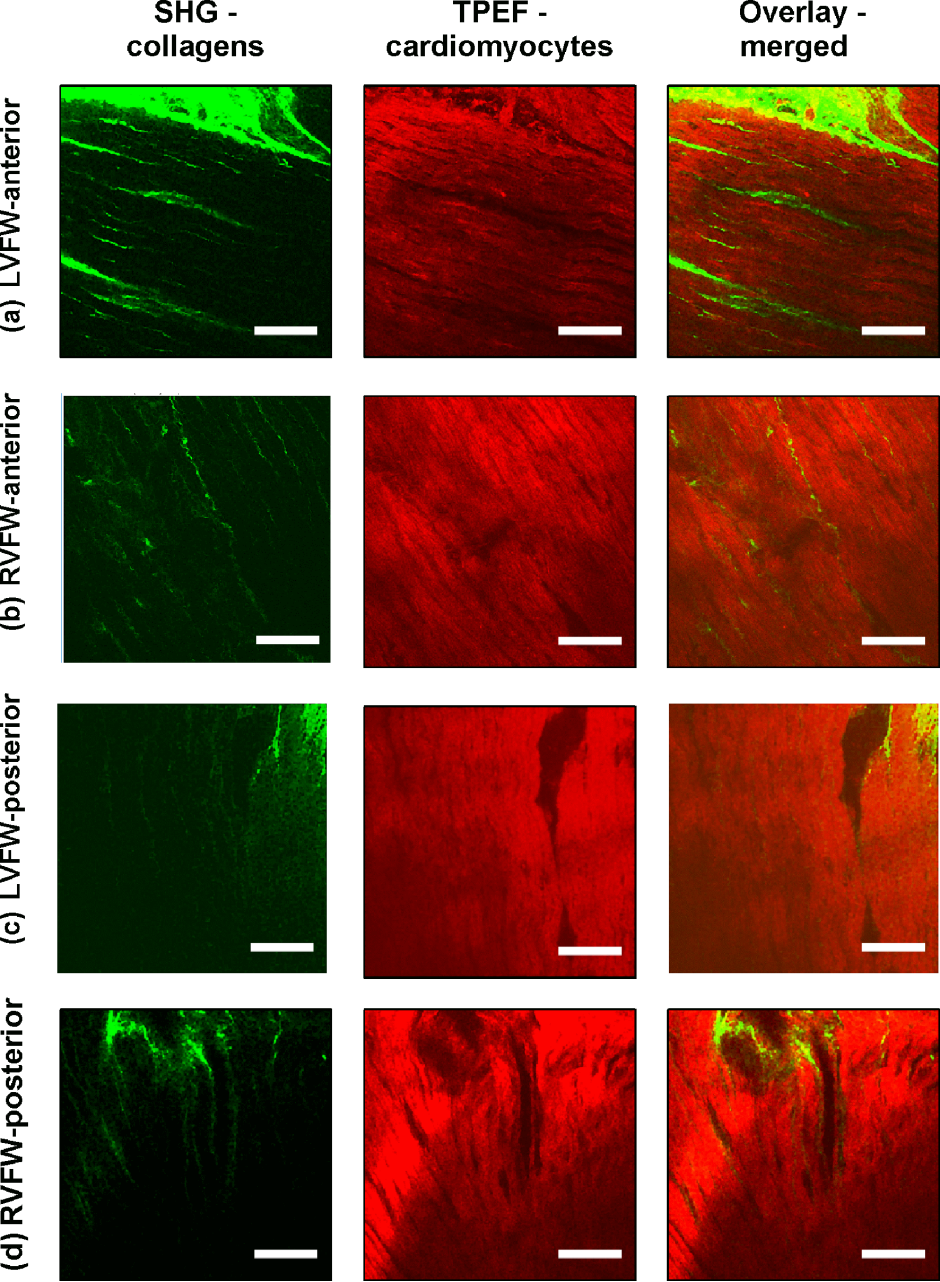
In-plane TPEF/SHG images, with the SHG-channel (green) identifying the collagen fibril distribution, and the TPEF-channel (red) the cardiomyocyte. Both channels were merged to demonstrate the collagen-cardiomyocyte overlapping. Scale bar = 100 *µ*m.

**Figure 3:**
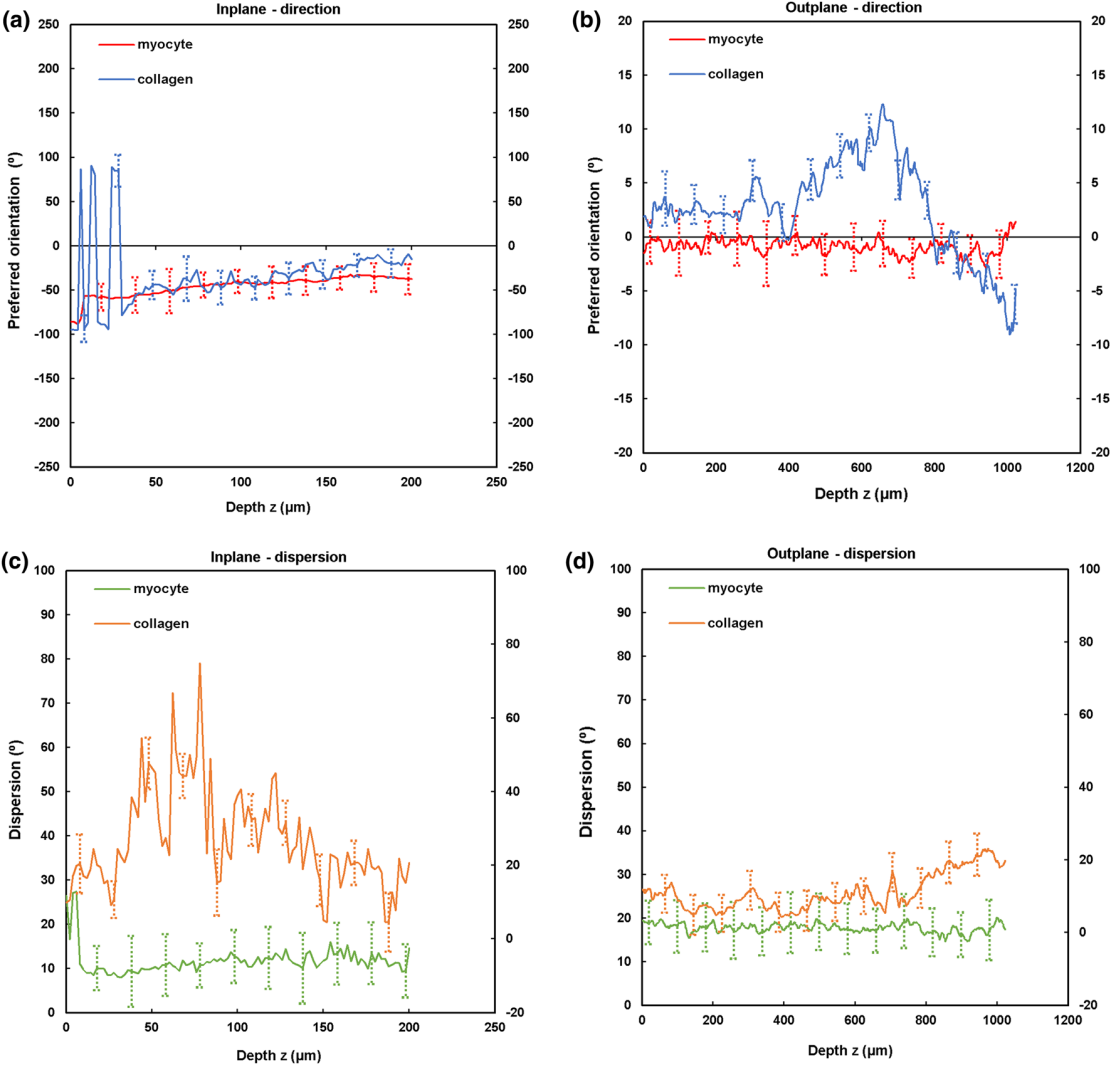

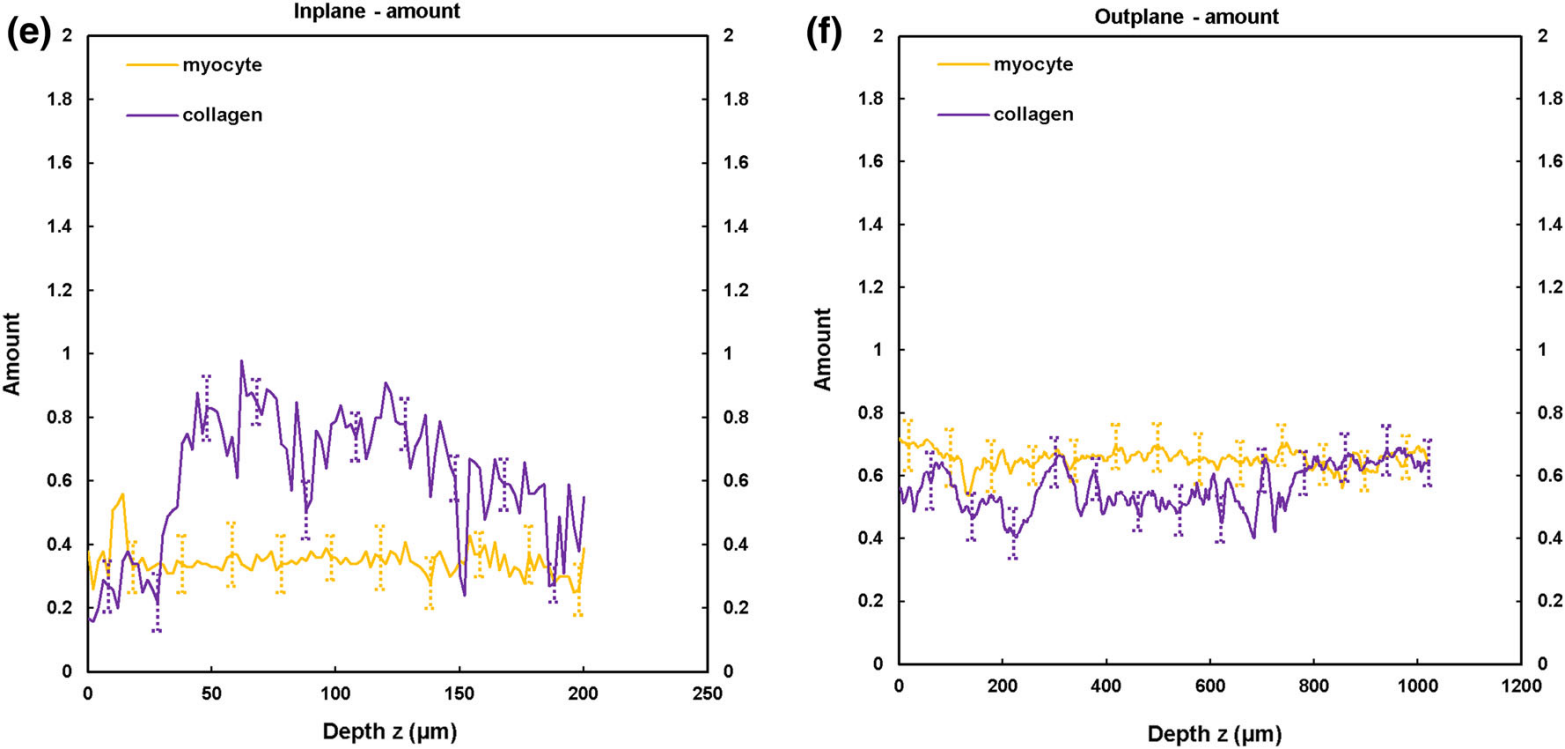
TPEF/SHG microscopy was used to quantify the preferred orientation of the cardiomyocyte and collagen fibril microstructural parameters in the anterior LVFW (*n* = 5). (a) in-plane preferred orientation; (b) out-plane preferred orientation. (c) in-plane dispersion; (d) out-plane dispersion. (e) in-plane amount; (f) out-plane amount. In-plane and out-plane image-stacks were acquired through the depth of 200-and 1022 *µ*m respectively.

**Figure 4:**
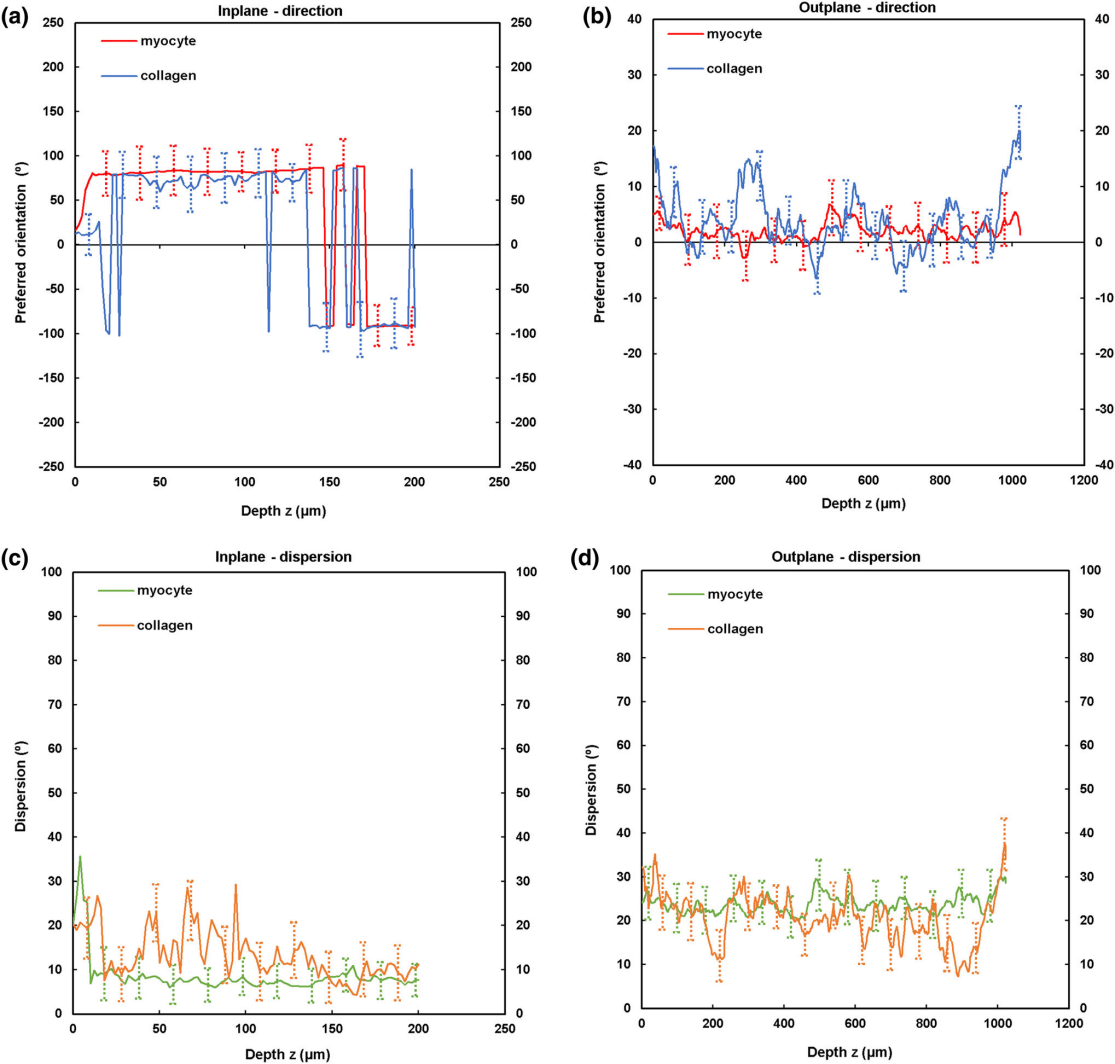

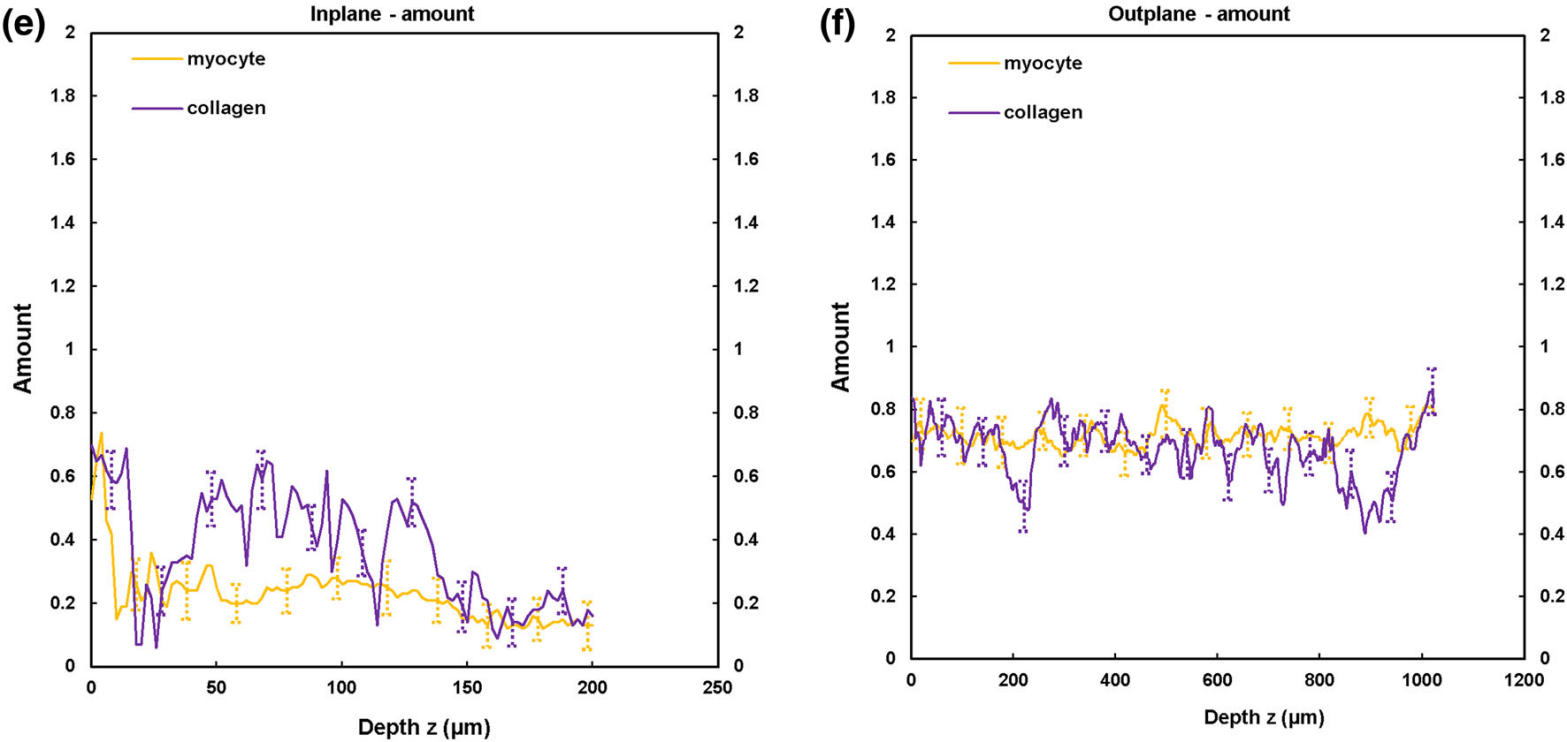
TPEF/SHG microscopy was used to quantify the preferred orientation of the cardiomyocyte and collagen fibril microstructural parameters in the anterior RVFW (*n *= 5). (a) in-plane preferred orientation; (b) out-plane preferred orientation. (c) in-plane dispersion; (d) out-plane dispersion. (e) in-plane amount; (f) out-plane amount. In-plane and out-plane image-stacks were acquired through the depth of 200-and 1022 *µ*m respectively.

**Figure 5:**
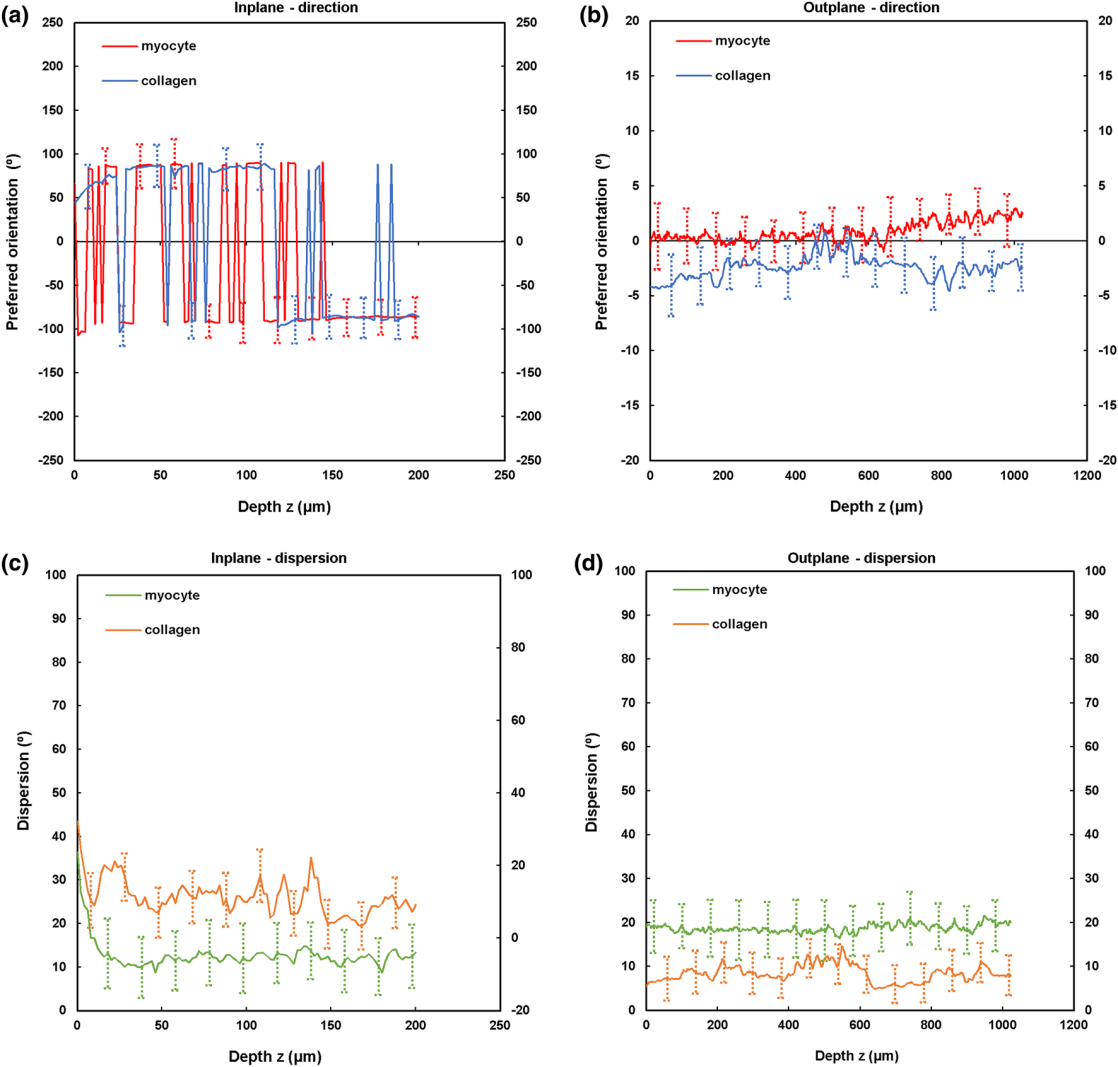

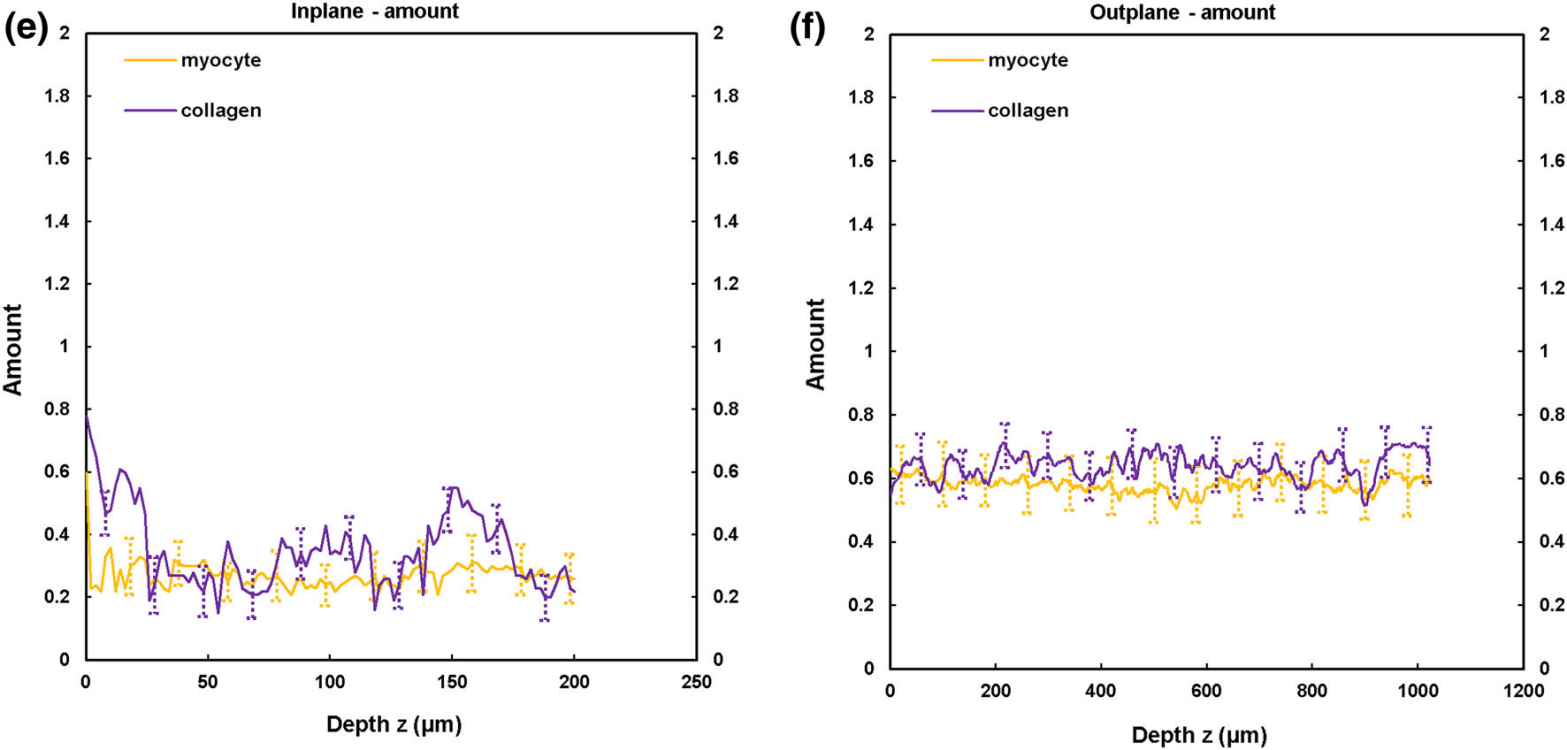
TPEF/SHG microscopy was used to quantify the preferred orientation of the cardiomyocyte and collagen fibril microstructural parameters in the posterior LVFW (*n *= 5). (a) in-plane preferred orientation; (b) out-plane preferred orientation. (c) in-plane dispersion; (d) out-plane dispersion. (e) in-plane amount; (f) out-plane amount. In-plane and out-plane image-stacks were acquired through the depth of 200-and 1022 *µ*m respectively.

**Figure 6:**
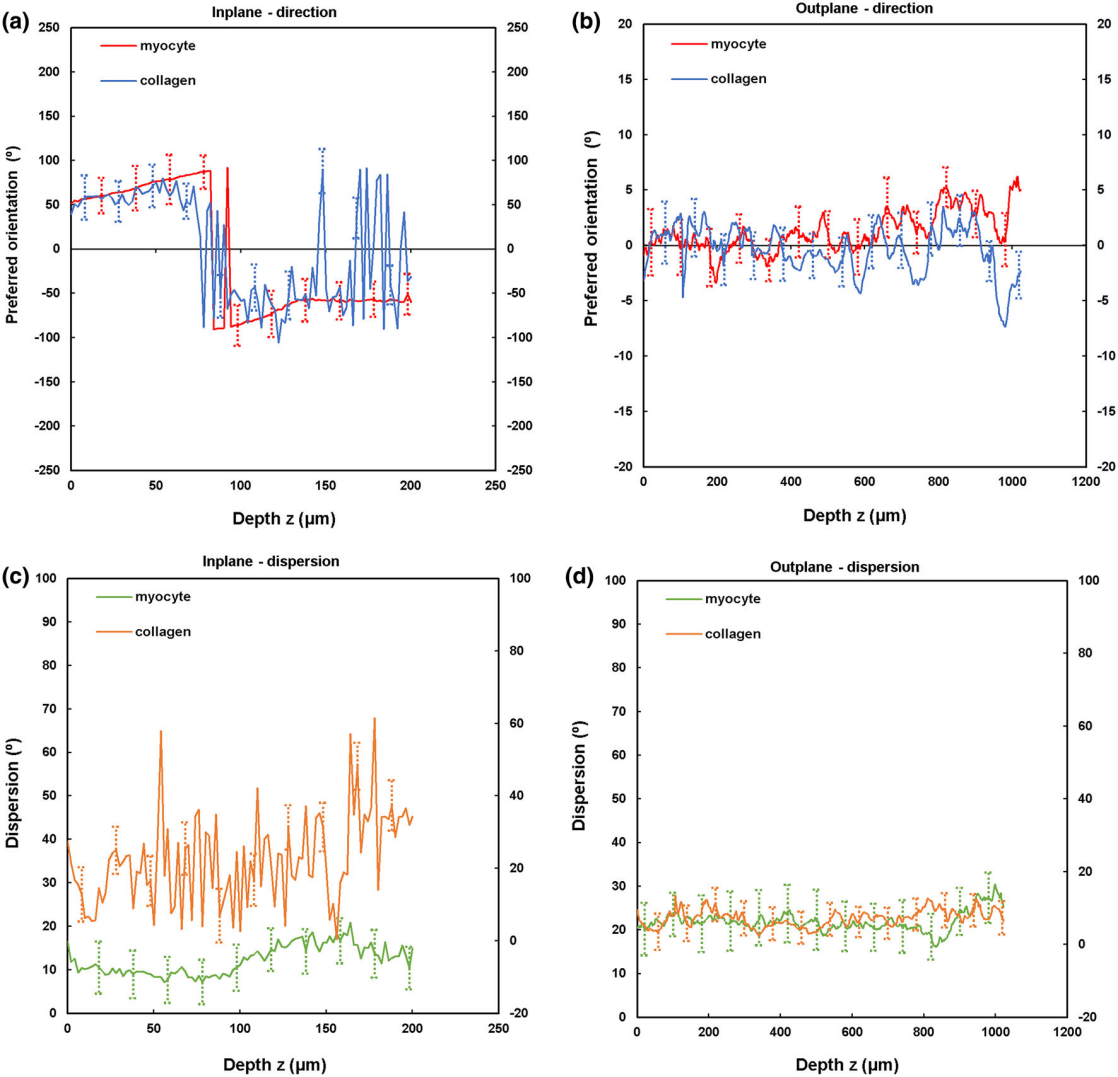

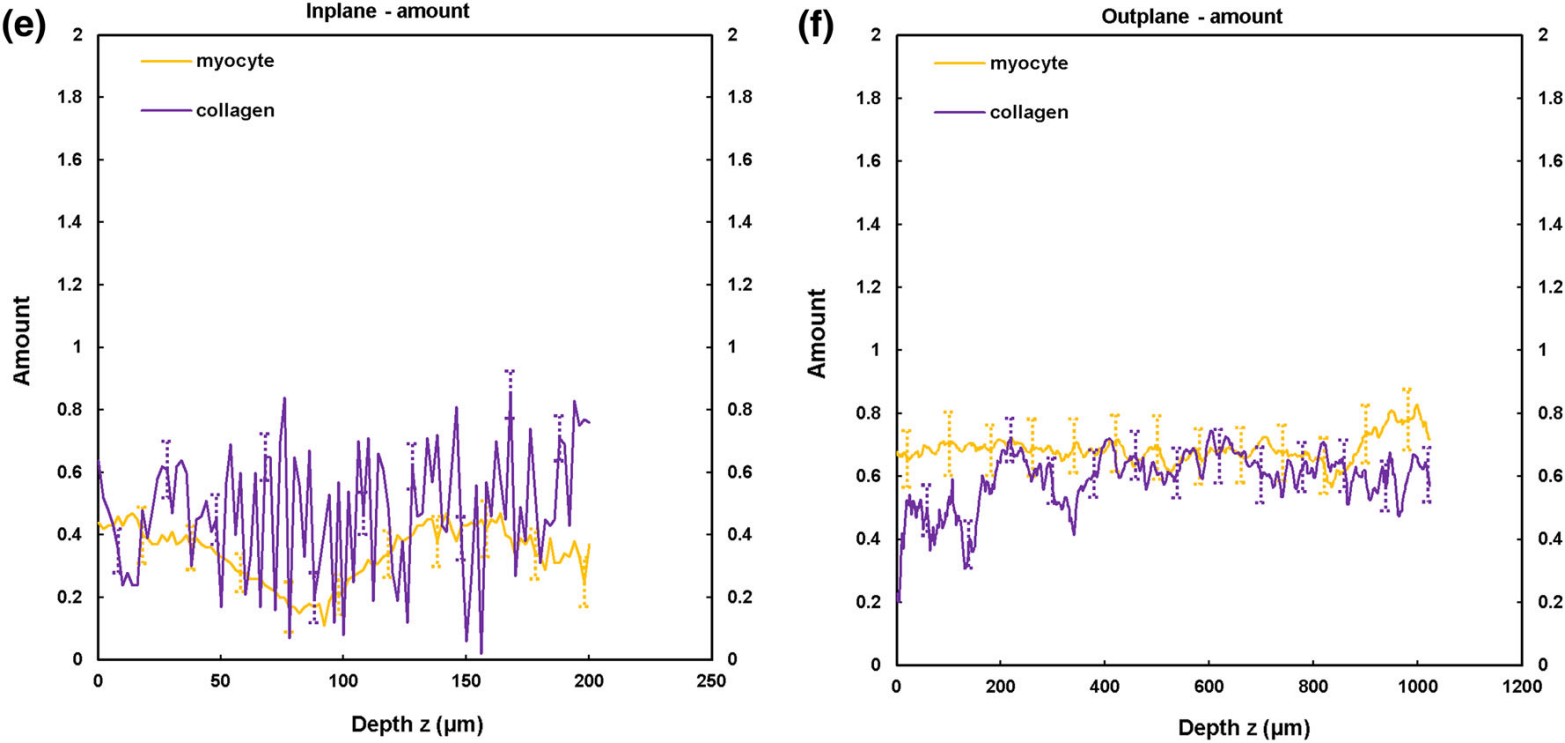
TPEF/SHG microscopy was used to quantify the preferred orientation of the cardiomyocyte and collagen fibril microstructural parameters in the posterior RVFW (*n *= 5). (a) in-plane preferred orientation; (b) out-plane preferred orientation. (c) in-plane dispersion; (d) out-plane dispersion. (e) in-plane amount; (f) out-plane amount. In-plane and out-plane image-stacks were acquired through the depth of 200-and 1022 *µ*m respectively.

TPEF/SHG channels were used to to identify the cardiomyocytes and collagen fibril distribution within the anterior and posterior aspects of the LVFW and RVFW. The SHG channel, visualized in green, identified the collagen fibril distribution, whilst the TPEF channel in red, highlighted cardiomyocyte distribution (Fig. 2). Merging the two channels enabled measuring the in-plane and out-plane cardiomyocyte and collagen fibril preferred orientation direction (Figs. 3a, 3b, 4a, 4b, 5a, 5b and 6a, 6b), angular dispersion (Figs. 3c, 3d, 4c, 4d, 5c, 5d and 6c, 6d) and amount parameters (Figs. 3e, 3f, 4e, 4f, 5e, 5f and 6e, 6f), through the anterior and posterior aspects of the LVFW and RVFW.

##### LVFW

**Figure 7:**
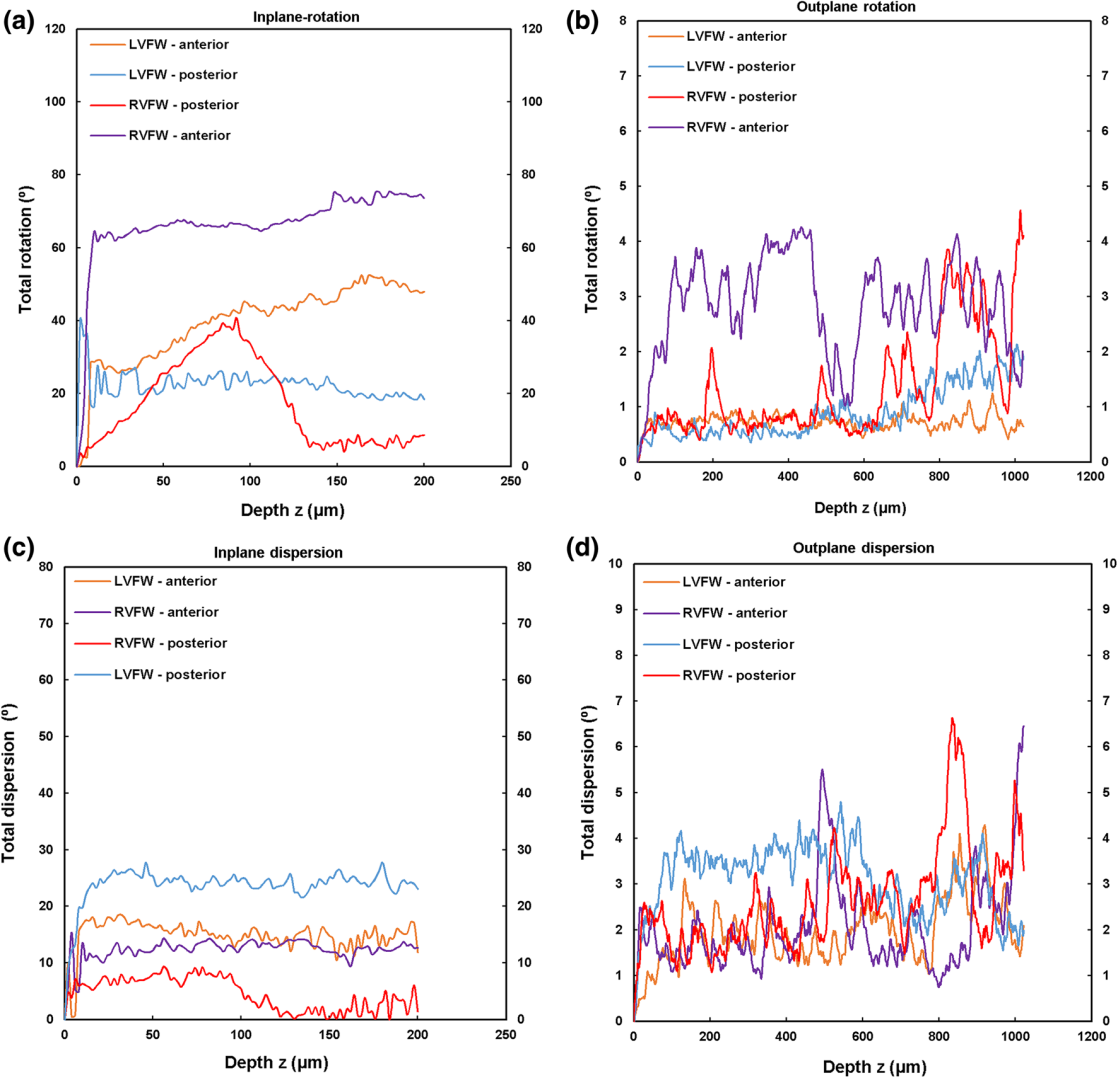
TPEF/SHG microscopy was used to quantify the extent of in-plane and out-plane cardiomyocytes rotation and dispersion in the anterior and posterior aspects of LVFW and RVFW. (a) in-plane rotation; (b) out-plane rotation. (c) in-plane dispersion; (d) out-plane dispersion. In-plane and out-plane image-stacks were acquired through the depth of 200-and 1022 *µ*m respectively.

**Figure 8:**
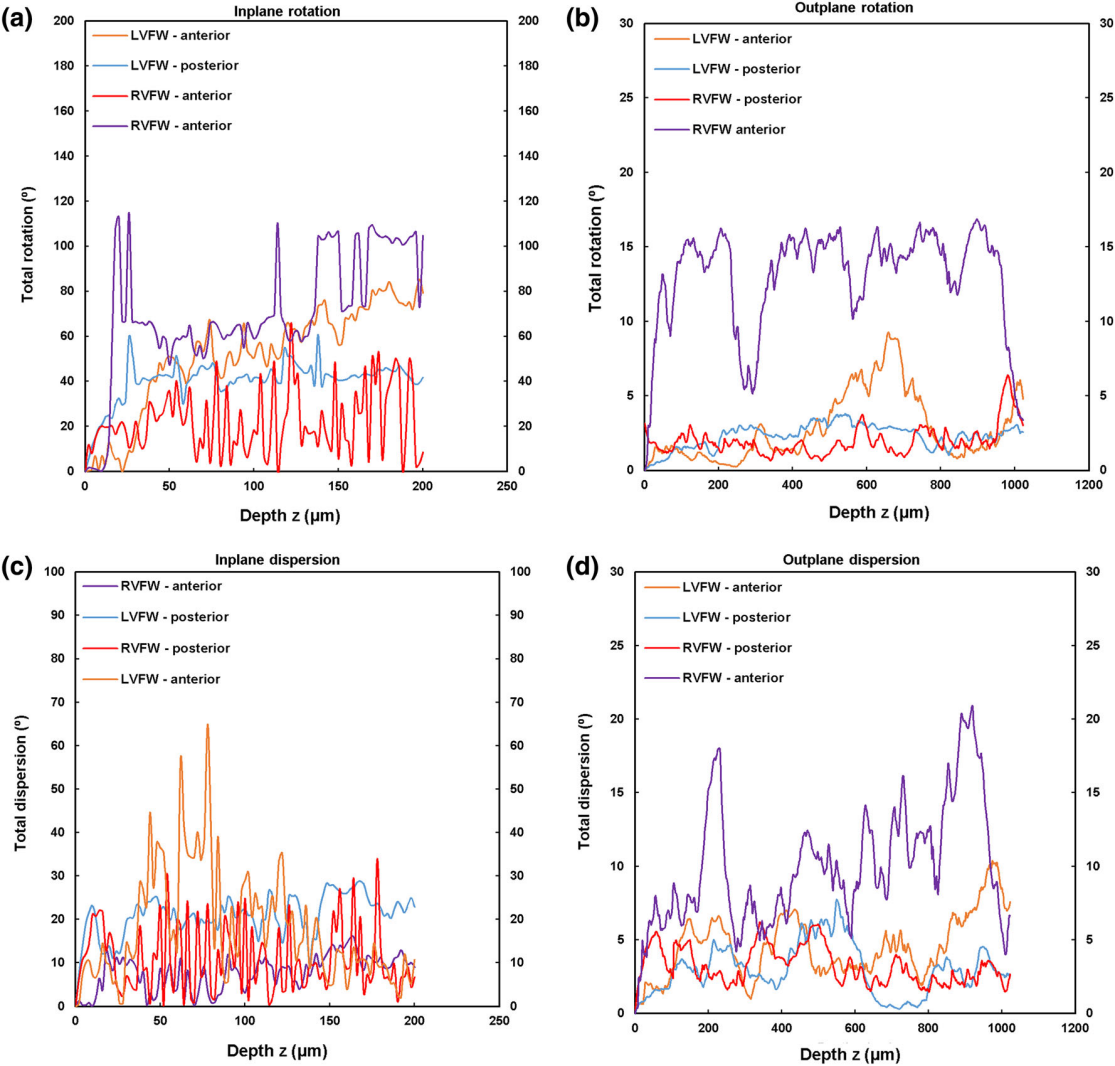
TPEF/SHG microscopy was used to quantify the extent of in-plane and out-plane collagen fibrils rotation and dispersion in the anterior and posterior aspects of LVFW and RVFW. (a) in-plane rotation; (b) out-plane rotation. (c) in-plane dispersion; (d) out-plane dispersion. In-plane and out-plane image-stacks were acquired through the depth of 200-and 1022 *µ*m respectively.

Cardiomyocytes and collagen fibrils exhibited a significantly greater in-plane rotation (39.3° and 51.1° respectively) in the anterior wall, than in the posterior wall (22.3° and 40.2° respectively) (Figs. 7a, 8a and Table 2). A similar trend was also evident when considering the in-plane ‘amount’, with the anterior wall recording 0.35 vs. 0.27 (posterior wall) for cardiomyocytes, and 0.61 vs. 0.35 for collagen fibrils, anteriorly and posteriorly respectively (Table 2). In-plane dispersion was significantly greater for both cardiomyocytes (23.7° vs. 14.8°) and collagen fibrils (20.9° vs. 17.5°) (Figs. 7c, 8c and Table 2).

**Table 2:** In-plane cardiomyocytes and collagen fibrils rotation, dispersion and amount through the LVFW and RVFW, across 200 *µ*m depth of the anterior (A) and posterior (P) aspects.

	Rotation (°)	Dispersion (°)	Amount
Cardiomyocytes			
LVFW (A)	39.3 ± 5.3^a,b^	14.8 ± 3.0^a,b^	0.35 ± 0.04^a,b^
RVFW (A)	66.2 ± 10.6^a,b^	12.3 ± 2.0^a,b^	0.23 ± 0.05^a,b^
LVFW (P)	22.3 ± 9.2^a,b^	23.7 ± 1.5^a,b^	0.27 ± 0.09^a,b^
RVFW (P)	17.2 ± 3.2^a,b^	4.5 ± 2.3^a,b^	0.34 ± 0.05^a,b^
Collagens			
LVFW (A)	51.1 ± 3.8^a,b^	17.5 ± 2.8^a,b^	0.61 ± 0.09^a,b^
RVFW (A)	70.5 ± 6.5^a,b^	7.80 ± 4.2^a,b^	0.37 ± 0.1^a,b^
LVFW (P)	40.2 ± 2.9^a,b^	20.9 ± 1.8^a,b^	0.35 ± 0.08^a,b^
RVFW (P)	22.1 ± 6.2^a,b^	10.8 ± 3.1^a,b^	0.48 ± 0.07^a,b^

Results are expressed as mean ± SD

^a^One-way analysis of variance (ANOVA) revealed statistical significance between the anterior (A) and posterior (P) aspects *within* the ventricle *p* < 0.05

^b^One-way analysis of variance (ANOVA) revealed statistical significance between the equivalent regions *across* the ventricles *p* < 0.05

The out-plane microstructure differs, with rotation in the posterior aspect being greater than the anterior aspect for cardiomyocytes (0.90° vs. 0.72°), though this trend was reversed for collagen (2.3° vs. 2.8° respectively) (Figs. 7b, 8b and Table 3). Cardiomyocyte out-plane amount was greatest anteriorly (0.65 vs. 0.58), whilst collagen fibril amount was greatest posteriorly (0.64 vs. 0.56) (Table 3). Out-plane cardiomyocyte dispersion was greatest in the posterior aspect (3.13°) vs. the anterior aspect (1.96°) for cardiomyocytes, whilst the anterior aspect had greater collagen fibril dispersion (4.5° vs. 2.9°) (Figs. 7d, 8d and Table 3). All differences were statistically significant (*p* < 0.05).

**Table 3:** Out-plane cardiomyocytes and collagen fibrils rotation, dispersion and amount through the LVFW and RVFW, across 1022 *µ*m depth in the anterior (A) and posterior (P) aspects.

	Rotation (°)	Dispersion (°)	Amount
Cardiomyocytes			
LVFW (A)	0.72 ± 0.04^a,b^	1.96 ± 0.09^a,b^	0.65 ± 0.03^a,b^
RVFW (A)	2.85 ± 0.03^a,b^	2.05 ± 0.04^a,b^	0.72 ± 0.07^a,b^
LVFW (P)	0.90 ± 0.02^a,b^	3.13 ± 0.06^a,b^	0.58 ± 0.02^a,b^
RVFW (P)	1.34 ± 0.025^a,b^	2.63 ± 0.04^a,b^	0.69 ± 0.06^a,b^
Collagens			
LVFW (A)	2.8 ± 0.07^a,b^	4.5 ± 0.03^a,b^	0.56 ± 0.04^a,b^
RVFW (A)	13 ± 0.01^a,b^	10 ± 0.08^a,b^	0.67 ± 0.02^a,b^
LVFW (P)	2.3 ± 0.04^a,b^	2.9 ± 0.05^a,b^	0.64 ± 0.03^a,b^
RVFW (P)	1.9 ± 0.08^a,b^	3.2 ± 0.06^a,b^	0.60 ± 0.04^a,b^

Results are expressed as mean ± SD

^a^One-way analysis of variance (ANOVA) revealed statistical significance between the anterior (A) and posterior (P) aspects *within* the ventricle *p* < 0.05

^b^One-way analysis of variance (ANOVA) revealed statistical significance between the anterior (A) and posterior (P) aspects *across* the ventricles *p* < 0.05

##### RVFW

In-plane cardiomyocyte rotation was greater in the anterior wall (66.2° vs. 17.2°) and was also significantly greater than in the equivalent LVFW tissue (Fig. 7a and Table 2). Collagen fibril rotation was again greatest in the anterior vs. posterior (70.5° vs. 22.1°), and significantly greater than the LVFW, whilst the posterior wall in-plane rotation was less than in the comparable LVFW tissue (Fig. 8a and Table 2). Cardiomyocyte in-plane dispersion was higher in the anterior wall than the posterior wall (12.3° and 4.5° respectively), though both were lower than that in the LVFW (Fig. 7c and Table 2). The posterior wall had greater collagen fibril dispersion (10.8° vs. 7.8°), though both were less than the LVFW (Fig. 8c and Table 2). In-plane cardiomyocyte amount was greater in the posterior aspect (0.34 vs. 0.23), with the anterior surface having less than the LVFW, whilst the posterior surface was greatest in the RVFW (Table 2). The anterior RVFW had lower collagen fibril amount (0.37) than the posterior aspect (0.48), with the former being less and the latter greater, than the LVFW (Table 2).

There is again little out-plane rotation of cardiomyocytes, though the anterior (2.85°) is greater than the posterior aspect (1.34°) (Fig. 7b and Table 3). Both are significantly greater than the LVFW (Table 3). Out-plane collagen rotation is high in the anterior aspect (13°), vs. 1.9° in the posterior aspect (Fig. 8b and Table 3). The former is greater than, and the latter less than, the equivalent LVFW measures (Table 3). Cardiomyocyte out-plane dispersion is slightly greater in the posterior vs. anterior wall (2.63° vs. 2.05°) (Fig. 7d and Table 3). The anterior aspect is greater than, though the posterior aspect less than, the RVFW (Table 3). Collagen fibril dispersion is greater in the anterior aspect (10° vs. 3.2°), and both are greater than in the LVFW (Fig. 8d and Table 3). Out-plane cardiomyocyte amount is marginally greater in the anterior wall (0.72) than the posterior wall (0.69), with both greater than the equivalent LVFW (Table 3). Out-plane collagen fibril amount is greater in the anterior wall than the posterior wall (0.67 vs. 0.60), with the former greater than, and the latter less than, the LVFW (Table 3).

## Discussion

These data describe the 1-day old heart microstructure and how it differs between the left and right ventricle, and the anterior and posterior walls. These differences provide a valuable insight into the newborn heart, as it begins to grow and develop in response to a new oxygenation supply and increasing physiological demands.

### Anterior and Posterior LVFW

Gross cardiomyocyte fibre orientation was quantified using FA, *via* DT-MRI (Fig. 1). The equator demonstrated significantly greater fibre orientation and density than the basal or apical regions (Supplementary Fig. 2). A qualitative assessment of alignment revealed that the anterior fibres were predominantly horizontal, whereas those on the posterior surface were more diagonally-aligned (Fig. 1).

In-plane cardiomyocyte rotation was greatest in the anterior wall, which had greater gross curvature when compared to the posterior surface (Fig. 7a and Table 2). It may be that this rotation contributes to increasing the contractile strength of the anterior wall, and the overall LVFW twisting, during contraction.^29^,^30^ Out-plane cardiomyocyte rotation in both the anterior and posterior LVFW is negligible by comparison (Fig. 7b and Table 3). In-plane cardiomyocyte dispersion was relatively high in both walls, which may imply similar stiffness in the *x*- and *y*-axes, as seen in previous biomechanical analysis.^11^ There was greater dispersion in the posterior surface (Fig. 7c and Table 2), which coincided with the least difference in biomechanical behaviour in the *x*- and *y*-axes.^11^ Out-plane dispersion was again significantly less (Fig. 7d and Table 3). This stark difference in rotation and dispersion in-plane and out-plane, suggests the cardiomyocyte fibres are organised within laminar sheets.^28^

Collagen fibrils exhibited greater in-plane rotation in the anterior and posterior LVFW than the cardiomyocytes (Figs. 7a, 8a and Table 2). Again, it is presumed this ensures a relatively high stiffness, especially in the anterior surface. Whilst the in-plane dispersion was more consistent across the two surfaces than the cardiomyocytes (Figs. 7c, 8c and Table 2), the greater dispersion posteriorly would provide further evidence that it will exhibit a similar bio-mechanical response when loaded in either a mean-fibre or cross-fibre direction. This in-plane dispersion of interstitial collagen provides myocardium structural stability, organising the cardiomyocyte architecture into organised, sheet layers.^4^,^36^

### Anterior and Posterior RVFW

Macroscopic assessment demonstrated some structural consistency with the LVFW (Fig. 1). FA and density was again greater in the equatorial region than the base and apex, with each also demonstrating significantly greater FA than the equivalent LVFW region (Supplementary Fig. 2 and Table 1). Unlike the LVFW, both the anterior and posterior cardiomyocytes were horizontally aligned (Fig. 2).

The anterior aspect of the RVFW had fourfold greater cardiomyocyte rotation than the posterior wall (vs. c. twofold for the LVFW), which would again imply a focus on achieving greater contractile strength (Fig. 7a and Table 2). The anterior fibres also had greater rotation than those in the LVFW (Fig. 7a and Table 2). This would all contribute to the RVFW demonstrating markedly different biomechanical behaviour when loaded in the cross-fibre and mean-fibre directions,^11^ vs. the comparable performance of the LVFW. Out-plane rotation again appears negligible vs. the equivalent in-plane measures, with such a difference again indicating that these fibres are likely organised within laminar sheets (Fig. 7b and Table 3).^4^,^36^ In-plane cardiomyocyte dispersion was low in the posterior wall, whilst the anterior wall measure was similar to the equivalent RVFW region (Fig. 7c and Table 2). In-plane and out-plane collagen rotation was highest of all measures in the anterior RVFW, presumably contributing to the previously reported stiffer biomechanical response (Figs. 8a, 8b, Tables 2 and 3).^11^

### Porcine Model

This neonatal tissue is derived from a 1-day old porcine model. Whilst this animal surrogate has been commonly used in similar studies,^2^,^3^,^33^ there are anatomical differences with the human heart that have been described in detail elsewhere.^9^ The 1-day old heart is at the start of a rapid developmental phase, accommodating a shift in oxygen supply from the placenta to the lungs and also responding to the increasing physiological demands of the neonate.

The data presented here provides an early snap-shot into this development, highlighting key differences that already exist in the right and left ventricle, anteriorly and posteriorly. These data will be relevant to scientists, engineers and mathematicians working within the field of neonatal cardiac mechanics and may enable progression in areas including CHD. Further work is planned to investigate how the cardiac tissue grows and remodels beyond the neonatal stage.

## Electronic supplementary material

Below is the link to the electronic supplementary material.

Supplementary material 1 (TIFF 7146 kb). The anterior (**a**) and posterior (**b**) aspects of the one-day-old neonatal porcine heart. The helical cardiomyocyte architecture of neonatal porcine heart before being processed; anterior view (**c**) and posterior view (**d**). The cardiomyocyte architecture of neonatal porcine heart after being processed; anterior view (**e**) and posterior view (**f**). The ellipsoids regions represent the undesirable tracks removed to obtain the required heart profile. Scale bar = 8 mm

Supplementary material 2 (TIFF 3747 kb). The anterior and posterior aspects of the one-day-old neonatal porcine heart. Fibre bundles were coloured to demonstrate the mean-fibre orientation, using 50% fibre density; anterior view (**a**) and posterior view (**b**). 25% fibre density; anterior view (**c**) and posterior view (**d**). 15% fibre density; anterior view (**e**) and posterior view (**f**) respectively
