# Supplementary figures and images for: Region-Specific Microstructure in the Neonatal Ventricles of a Porcine Model
Source: Ann Biomed Eng. 2018 Jul 16;46(12):2162–76. doi: 10.1007/s10439-018-2089-4 (PMC6267669; doi:10.1007/s10439-018-2089-4)

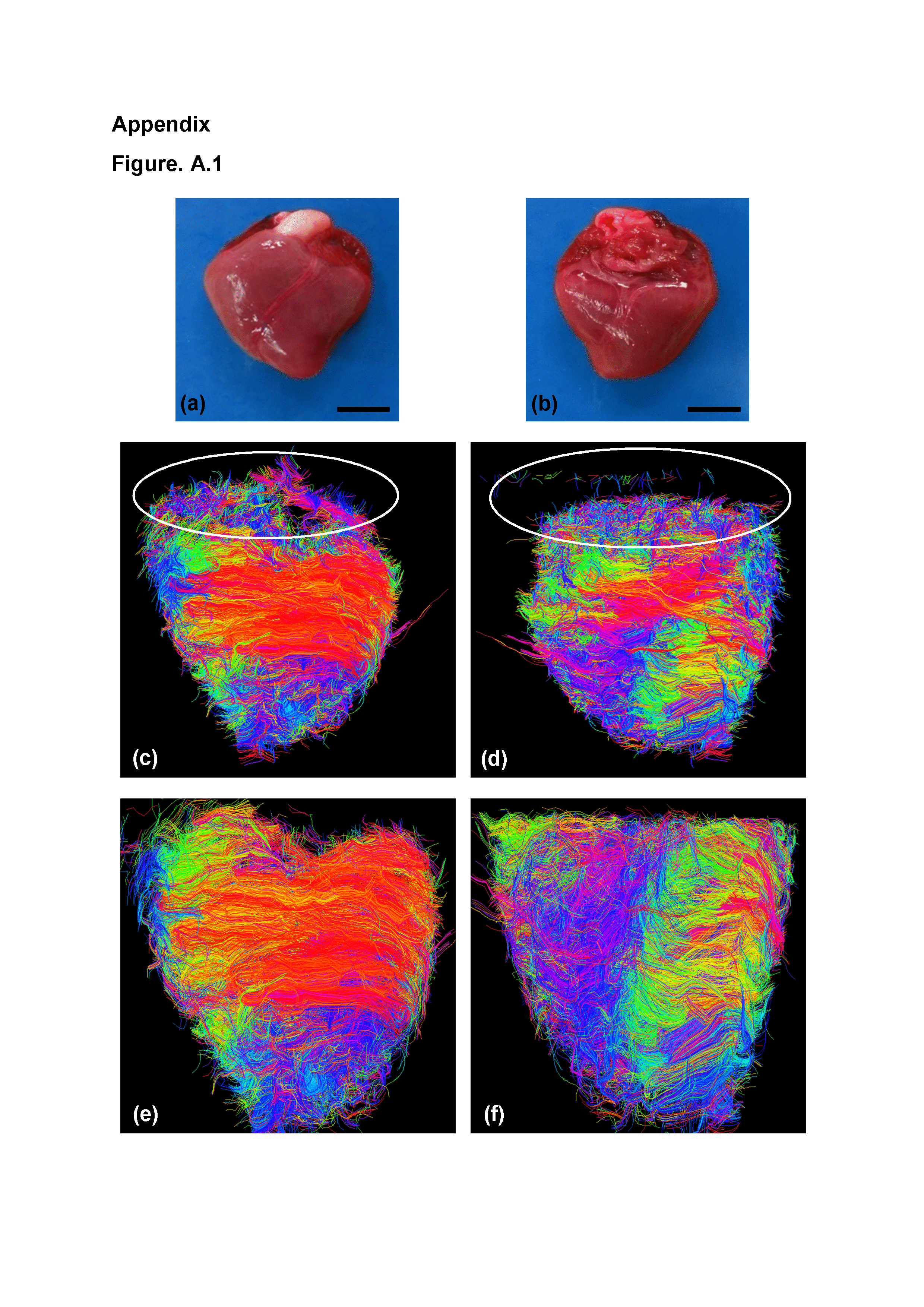

Supplement: Supplementary file 1 — Supplementary material 1 (TIFF 7146 kb). The anterior (a) and posterior (b) aspects of the one-day-old neonatal porcine heart. The helical cardiomyocyte architecture of neonatal porcine heart before being processed; anterior view (c) and posterior view (d). The cardiomyocyte architecture of neonatal porcine heart after being processed; anterior view (e) and posterior view (f). The ellipsoids regions represent the undesirable tracks removed to obtain the required heart profile. Scale bar = 8 mm [file 10439_2018_2089_MOESM1_ESM.tif]
